# Sustainable valorization of co-products from asparagus cultivation by obtaining bioactive compounds

**DOI:** 10.3389/fpls.2023.1199436

**Published:** 2023-07-13

**Authors:** Isabel Viera Alcaide, Amel Hamdi, Rafael Guilleín-Bejarano, Ana Jiménez-Araujo, Rocío Rodríguez-Arcos

**Affiliations:** Phytochemicals and Food Quality Group, Instituto de la Grasa, Consejo Superior de Investigaciones Científicas (CSIC), Sevilla, Spain

**Keywords:** asparagus, by-products, crops, bioactive extracts, flavonoids, saponins

## Abstract

Asparagus cultivation generates every year a significant amount of by-products that consist of root and frond. Leaving these residues on the fields after harvesting negatively affects the following asparagus crops, since they release autotoxic (allelopathic) substances into the soil, whose accumulation causes that asparagus yields gradually decrease over the years, becoming an unprofitable crop in a period of about 10 to 15 years. This phenomenon is known as decay and forces the entire asparagus plantation to be lifted (abandoned). On the other hand, once a certain plantation has been lifted, it is not profitable to immediately re-plant new asparagus plants, since the yields that are achieved are never more than half of normal ones. It is necessary to wait an average of 4 or 5 years before replanting asparagus in these lands. This phenomenon is known as the replanting problem, and causes the need to continually search for new land for growing asparagus. Another added problem for farmers is that the elimination of those plant residues from asparagus cultivation entails significant economic costs. For all these reasons, it is essential to seek alternatives for the management of that waste that improve the sustainability of the crop within the scope of the circular economy. In this context, this work proposes the valorization of asparagus by-products by obtaining bioactive compounds. Main objectives of the present work include: i) phytochemical analyses of asparagus fronds and roots; ii) obtaining bioactive extracts, with distinct technological and nutritional functionalities, by using an environmentally sustainable extraction process, easy to implement in the practice of a food industry and with methods compatible with food use. Characterization of asparagus by-products shown that fronds had an average flavonoid content of 2.637 ± 0.014 g/Kg fresh weight, which is up to 5-6 times higher than that of the spears; and roots contained up to 10 times more saponins (2.25 g/Kg fresh weight), which were accompanied by lower quantities of phenolic acids (368 mg/Kg fresh weight). Statistical analysis revealed that those phytochemical contents were mainly determined by location and phase of the vegetative cycle, whereas genetic factors did not significantly influence them. Based on the results of the present work, the proposal for the recovery and valorization of asparagus by-products is based on obtaining two bioactive extracts, the first being an antioxidant extract enriched in flavonoids, with an average yield of 10.7 g/Kg fresh frond and a flavonoid richness of 17%; and the second, a saponins extract with an average yield of 10.3 g/Kg fresh root and a richness of 51%. These natural extracts have great techno-functional potential in the agri-food industry and some of them are already being tested as additives in the preparation of soups, breads and meat products.

## 1 Introduction

World consumption of asparagus is around 10 million and the current interest of consumers in maintaining a healthy diet will contribute to maintaining a high demand for this appreciated product, which is in the top of antioxidant plant foods. China and Peru lead the world production of white and green asparagus respectively. Spain ranks fifth worldwide and second in Europe, behind Germany (FAO, 2021; Shahbandeh, 2021).

The latest data available shows that 68,000 tons of asparagus are produced in Spain per year; more than 45,000 tons come from Andalusia (Observatorio de precios y mercados, CAPADS, 2023). Taking into account that for each cultivated hectare approximately 6 tons of fronds are harvested each year, the total volume of this by-product is equivalent to that of asparagus production (45,000 tons). On the other hand, in one hectare between 30 and 40 tons of roots are accumulated and 10% of the crop is raised annually, so the total amount of rhizome is about 25,000 tons per year. The elimination of these plant residues represents a major environmental problem, influenced by different factors. The first is the soil contamination, mainly produced by the residues that are generated when asparagus plantations ceases to be productive and must be replaced. The common agricultural practice of incorporating the root and rhizome into the ground could be the main cause of the phenomenon known as “asparagus decay”, which consists of a loss of crop quality and yield that appears after a few (5-7) years of crop and it is maintained and accentuated over time (Schofield, 1991; Zandstra et al, 2013). Furthermore, the problem is intensified by the impossibility of replanting with new young asparagus plants on the same crop fields when the old ones are raised, which is known as “asparagus replant problem” (Morrison et al, 2011; Yergeau et al, 2006; Elena, 2007). This limits the commercial life of the plantation and generates a continuous search for new cultivation lands. On the other hand, there is risk of air pollution derived of the burning of fronds and roots, which is a practice increasingly used by farmers. In fact, if the plantation is in an integrated production regime, it is mandatory to eliminate the aerial part of the plant and its incorporation into the land is not allowed. However, the roots continue to leave them when the plantation is raised, since eliminating them would entail a cost that in most cases the farmer cannot assume. In addition, in cases where it is possible to take the waste to the landfill, its transport entails great economic and environmental costs.

The “asparagus decay” and “asparagus replant problem” are thought to be caused by a combination of biotic and abiotic stresses Grogan and Kimble (1959). The pathogen infection of *Fusarium* species that accumulate in the soil was reported to be the main biotic stress (Baayen et al, 2000; Elmer and Pignatello, 2011; Nahiyan et al, 2011; Brizuela et al, 2020). During the last years, many efforts have been made related to the development of fungicides for the control of *Fusarium* (Reid et al, 2002; Zandstra et al, 2013); and alternative methods, such as the addition of organic amendments (Baayen et al, 2000), biological control (Borrego-Benjumea et al, 2010) and development of bio-phytosanitary products (Rosado-Alvarez et al, 2014) are promising However, the mechanisms of action still need to be studied in depth to achieve the most effective formulations against *Fusarium*. Along with *Fusarium* infections, a*utotoxicity* of asparagus tissues has been reported to be the main abiotic stress related to decline and replantation problem. This is a particular type of allelopathy, caused by some compounds released from asparagus plants. Investigations about the nature of these allelochemicals have revealed that they are phenolics, mainly caffeic acid, which is the most abundant phenolic compound in asparagus roots (Miller et al, 1991). Other phenolics identified from root exudates, which can contribute to the inhibition of asparagus growth are trans-cinammic, coumaric and ferulic acid (Elmer and Pignatello, 2011; Kato-Noguchi et al, 2018; Liu and Matsubara, 2018). The contamination by phenolic compounds not only comes from the rhizome exudates during the productive years of the plantation, but also from the asparagus residues incorporated into the asparagus cultivation soils Hazebroek et al, 1989; Yang et al, 1982. Hence, flavonoids (mainly rutin) and caffeic acid, released to the soil from the fronds and roots of asparagus, may contribute to a great extension to the autotoxic effects of asparagus cultivation (Hartung et al., 1990; Yeasmin et al., 2013; Noperi-Mosqueda et al, 2020). During the last years, many efforts have been made related to the improvement of cultivars that prevent the release of phytotoxic substances and to the control of soil pathogens. However, toxic asparagus residues remain a major stressor, in replant problem and significantly decrease field longevity and yield potential (Elmer, 2018). Those asparagus by-products that initially represent economic and environmental problems are full of interesting compounds that it is worthy to recover and exploit.

Previous studies on asparagus composition revealed that main phytochemicals are flavonoids (Fuentes-Alventosa et al, 2007; Guillén et al, 2008) and saponins (Vazquez-Castilla et al, 2013; Jaramillo-Carmona et al, 2017), which confer it a high added value. Thus, asparagus is among the plant products with the highest antioxidant capacity, which is mainly due to its flavonoid content (Vinson et al, 1998). Flavonoids generally occur in plants as glycosylated derivatives, asparagus contains significant amounts of glycosides derived from three aglycones, quercetin, kaempferol and isorhamnetin and the most abundant is quercetin-3-rhamnosyl glucoside, known as quercetin-rutinoside or rutin (Guillén et al., 2008). The antioxidant activity of asparagus is mostly attributed to rutin (Chin et al., 2002; Chin and Garrison, 2008), existing a high correlation between these two parameters (Drinkwater, 2013). Flavonoids, in addition to a high antioxidant capacity have antitumoral and antimicrobial activities (Cushine and Lamb, 2005). Saponins are a group of phytochemicals, present in numerous plant species, which are classified as triterpenic and steroidal according to the structure of their constituent aglycone (Oleszeck and Marston, 2000). The genus Asparagus is one of the few that contains steroid-type saponins that are distributed throughout different organs of the plant. Studies on steroidal saponins have focused on their pharmacological properties and, mainly, on their preventive and control role in various types of cancer, since these compounds have shown a high cytotoxic and cytostatic capacity against tumor cells (Podolak et al., 2010). The relevance of saponins in the sensory characteristics of asparagus has also been pointed out (Dawid and Hofmann, 2012). Those interesting compounds are located not only in the edible part, but also in the basal portion and peels that are discarded during asparagus processing (Fuentes-Alventosa et al, 2013). More recent investigations have led that by-products generated during cultivation represent even a better source of phytochemicals, since fronds contain five times more flavonoids than spears and roots up to 10 times more saponins (Viera et al, 2020). Most of the reported methods for the determination of asparagus phytochemicals involve their extraction with hydro-alcoholic solutions and their identification and quantification by HPLC-DAD-MS (Wang et al, 2003; Fuentes-Alventosa et al, 2007; Lee et al, 2010; Vazquez-Castilla et al, 2013). There have been also developed optimized procedures for the recovery of bioactive compounds from asparagus waste, based on those conditions that are commonly used for the industrial canning of plant foods. The treatment of asparagus by-product with hot water, in an industrial autoclave, allows obtaining aqueous extracts containing most soluble bioactive compounds from asparagus by-product (Guillén et al, 2008). From these evidences, the main objective of the present work is the valorization of asparagus cultivation co-products by obtaining extracts enriched in the different phytochemicals present in asparagus tissues, mainly flavonoids and saponins.

## 2 Materials and methods

### 2.1 Plant material

The samples investigated consisted of fronds (aerial part) and roots and rhizomes that are discarded during asparagus cultivation. The first are generated every year when the asparagus branches are cut after harvesting the spears, and the second, every 10-12 years when asparagus plantations are raised. As there are many asparagus plantations with different ages, each year it is the turn of several of them to be renewed. As there are many asparagus plantations with different ages, each year it is the turn of several of them to be renewed. Therefore, large quantities of the two types of co-products from asparagus cultivation are generated annually: roots and fronds.

The samples that have been investigated in the present work are listed in **Table 1** and consisted of 6 distinct cultivars of *A. officinalis L.*, collected in 2018 and 2019 from five different locations, four of them in the province of Cádiz and one in Granada. The five cultivars from Cadiz and the farms when they were collected were: *Herkolim from IFAPA* (HI), *Primens from IFAPA* (PI), *Alamo from La Negra* (AN), *Grande from Doña Blanca* (DB), *Atticus from Doña Blanca (A)* and *Grande from Manrique (GM)*; and the two cultivars from Granada were *Grande from Huétor-Tájar* (GHT) *and Triguero from* Huétor-Tájar (THT). The age of the plantations ranged from 2 to 14 years. The survey dates and the location of each farm are shown in **Table 1**. In each survey, whole units of plants were collected, including fronds and roots, which were later processed in the laboratory. Plants were sampled at three different stages in the vegetative cycle of the plant: June-July once the harvest of the spears is finished (1), September-October (2) and December-January (3). At this last point, the plants no longer showed green fronds and it was only possible to get root samples.

**Table 1:** Genetic and environmental characteristics of asparagus by-product samples.

Fronds								
Cultivar		Location		Plants Age		Harvest date		Sample code
Herkolim		IFAPA Chipiona, Cádiz (36.747561° N, -6.404574°)O)		4 years		21-06-2018		F-HI1
Primens		IFAPA Chipiona, Cádiz (36.747561° N, -6.404574° O)		4 years		21-06-2018		F-PI1
Álamo		Cortijo La Negra, Puerto Sta María, Cádiz (36.632665° N, -6.276930° O)		14 years		21-06-2018		F-AN1
Grande		Poblado Dña Blanca, Puerto Sta María, Cádiz (36.619731° N, -6.148597° O)		1 year		21-06-2018		F-GDB1
Áticus		Poblado Dña Blanca, Puerto Sta María, Cádiz (36.619731° N, -6.148597° O)		1 year		21-06-2018		F-ADB1
Herkolim		IFAPA Chipiona, Cádiz (36.747561° N, -6.404574°)O)		4 years		27-09-2018		F-HI2
Primens		IFAPA Chipiona, Cádiz (36.747561° N, -6.404574° O)		4 years		27-09-2018		F-PI2
Grande		Sonia Manrique, Puerto Sta María, Cádiz (36.632737° N, -6.258893° O)		10 years		27-09-2018		F-GM2
Grande		Poblado Dña Blanca, Puerto Sta María, Cádiz (36.619731° N, -6.148597° O)		1 year		27-09-2018		F-GDB2
Áticus		Poblado Dña Blanca, Puerto Sta María, Cádiz (36.619731° N, -6.148597° O)		1 year		27-09-2018		F-ADB2
Grande		Huétor Tájar, Granada (37.196870° N, -4.046830° O)		10 years		01-07-2018		F-GHT1
Triguero		Huétor Tájar, Granada (37.196870° N, -4.046830° O)		10 years		01-07-2018		F-THT1
Grande		Huétor Tájar, Granada (37.196870° N, -4.046830° O)		10 years		20-10-2018		F-GHT2
Triguero		Huétor Tájar, Granada (37.196870° N, -4.046830° O)		10 years		20-10-2018		F-THT2
Roots								
Cultivar		Location		Plants Age		Harvest date		Sample code
Herkolim		IFAPA Chipiona, Cádiz (36.747561° N, -6.404574°)O)		4 years		21-06-2018		R-HI1
Primens		IFAPA Chipiona, Cádiz (36.747561° N, -6.404574° O)		4 years		21-06-2018		R-PI1
Álamo		Cortijo La Negra, Puerto Sta María, Cádiz (36.632665° N, -6.276930° O)		14 years		21-06-2018		R-AN1
Grande		Poblado Dña Blanca, Puerto Sta María, Cádiz (36.619731° N, -6.148597° O)		1 year		21-06-2018		R-GDB1
Áticus		Poblado Dña Blanca, Puerto Sta María, Cádiz (36.619731° N, -6.148597° O)		1 year		21-06-2018		R-ADB1
Herkolim		IFAPA Chipiona, Cádiz (36.747561° N, -6.404574°)O)		4 years		27-09-2018		R-HI2
Primens		IFAPA Chipiona, Cádiz (36.747561° N, -6.404574° O)		4 years		27-09-2018		R-PI2
Grande		Sonia Manrique, Puerto Sta María, Cádiz (36.632737° N, -6.258893° O)		10 years		27-09-2018		R-GM2
Grande		Poblado Dña Blanca, Puerto Sta María, Cádiz (36.619731° N, -6.148597° O)		1 year		27-09-2018		R-GDB2
Áticus		Poblado Dña Blanca, Puerto Sta María, Cádiz (36.619731° N, -6.148597° O)		1 year		27-09-2018		R-ADB2
Herkolim		IFAPA Chipiona, Cádiz (36.747561° N, -6.404574°)O)		4 years		04-12-2018		R-GHI3
Primens		IFAPA Chipiona, Cádiz (36.747561° N, -6.404574° O)		4 years		04-12-2018		R-PI3
Grande		Huétor Tájar, Granada (37.196870° N, -4.046830° O)		10 years		01-07-2018		R-GHT1
Triguero		Huétor Tájar, Granada (37.196870° N, -4.046830° O)		10 years		01-07-2018		R-THT1
Grande		Huétor Tájar, Granada (37.196870° N, -4.046830° O)		10 years		20-10-2018		R-GHT2
Triguero		Huétor Tájar, Granada (37.196870° N, -4.046830° O)		10 years		20-10-2018		R-THT2
Grande		Huétor Tájar, Granada (37.196870° N, -4.046830° O)		10 years		10-01-2019		R-GHT3
Triguero		Huétor Tájar, Granada (37.196870° N, -4.046830° O)		10 years		10-01-2019		R-THT3

### 2.2 Chemicals and reagents

Authentic standards of caffeic acid, p-coumaric acid, t-ferulic acid and rutin (quercetin 3-O-rutinoside) were purchased from Sigma-Aldrich Quimica (Madrid, Spain). Kaempferol-3-O-rutinoside (nicotiflorin), isorhamnetin 3-O-rutinoside (narcissin), and isorhamnetin 3-O-glucoside were purchased from Extrasynthese (Genay, France). Protodioscin and shatavarin, were purchased from Chromadex Chemical Co. (Barcelona, Spain). Ethanol, formic acid (96%), and acetonitrile, HPLC grade, were purchased from Sigma Chemical Co. (St. Louis, MO, USA). All sample solutions were prepared using pure deionized water, which was obtained from a Milli-Q 50 system (Millipore Corporation, Bedford, MA, USA).

### 2.3 Ethanolic extracts

Ethanolic extraction was performed using the plant material (25 g) with 100 mL 96% EtOH. The samples were blended in a Thermomix Vorwerk, Model TM31 (Vorwerk, Spain), at maximum speed for 1 min, and then filtered through filter paper (Anoia, Barcelona, España). The residue was then extracted with 80% EtOH, in same conditions, and the filtrates were combined. Ethanolic extracts were stored at -20°C until HPLC analysis. All extractions were made in duplicate.

### 2.4 Aqueous extracts

The aqueous extractions of phytochemicals from fronds consisted of the treatment of 130 g of sample with 1 L of water, in an Selecta autoclave, Model Presoclave 75 (Selecta, Barcelona, Spain), at 121°C, for 1 hour. In the case of roots, 50 g of sample was extracted with 200 mL water, at 80°C, for 30 minutes. Solutions were filtered through filter paper (Anoia, Barcelona, España) and the aqueous extracts were stored at -20°C until HPLC analysis. All extractions were made in duplicate.

### 2.5 Fractionation and purification of functional extracts from asparagus by-product

**Figure 1:**
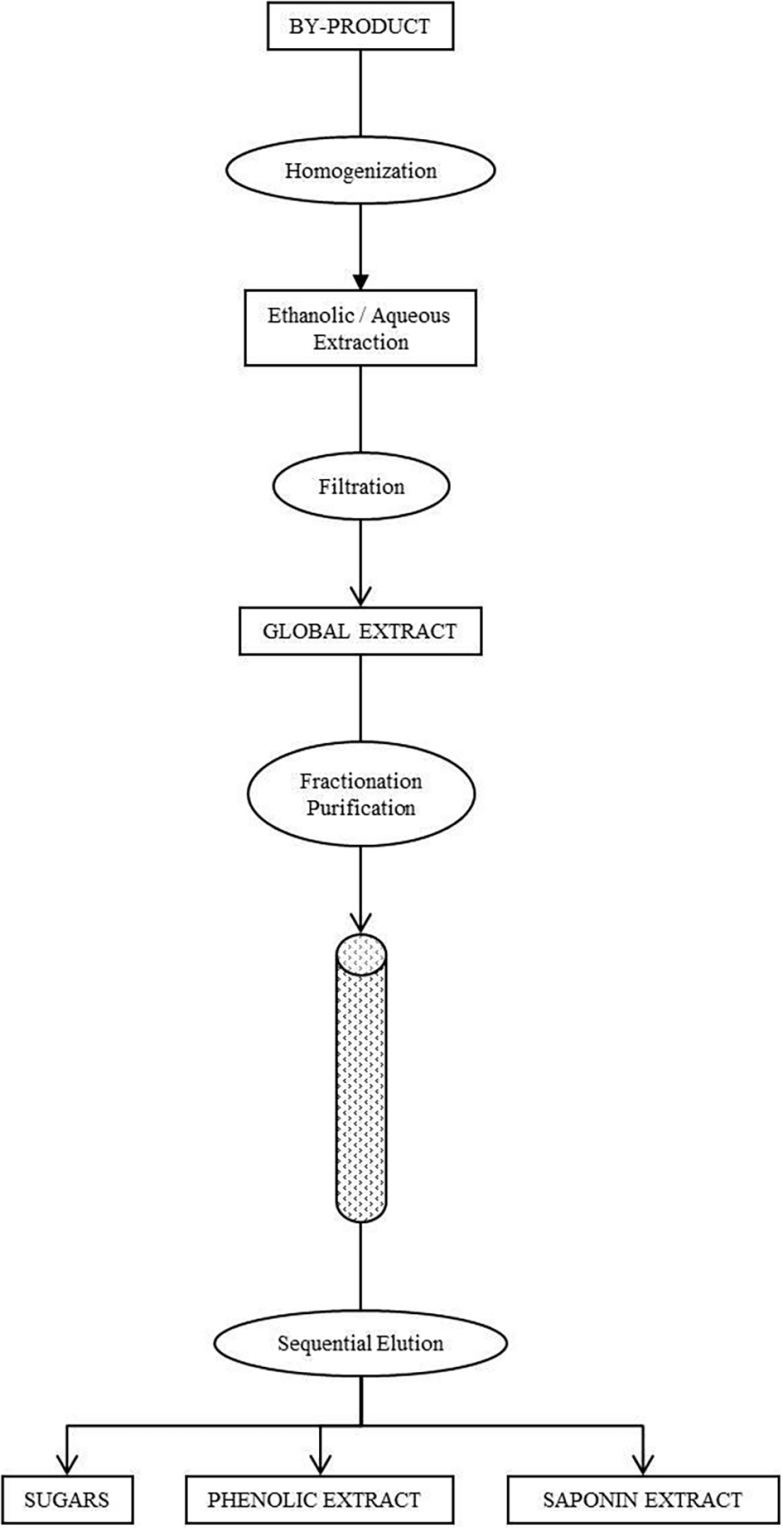
Procedure for the fractionation of global extracts from fronds and roots of asparagus.

Global extracts from fronds and roots, containing asparagus phytochemicals, mainly phenolic compounds and saponins, were fractionated and partially purified by chromatography in a column filled with Amberlite XAD polymeric adsorption resin (Rohm and Hass, España, S.A.). Bioactive compounds present in asparagus extracts were separated in base to their different polarity. The purification of the flavonoid extracts from the fronds was carried out as follows: after the injection of 1 L sample, the column was washed with 1 column volume of water, discarding this aqueous fraction that contains the sugars present in the global extract; and then sequentially with 2 column volumes of 40% ethanol aqueous solution (40% EtOH fraction). For roots, after the injection of 1 L sample, the column was washed with 1 column volume of water (aqueous fraction) and then sequentially with 2 column volumes of 20% ethanol aqueous solution (20% EtOH fraction) and 2 column volumes of 80% ethanol aqueous solution (80% EtOH fraction). The aqueous fraction contained sugars and oligosaccharides, whereas the phenols eluted with 20% ethanol and the saponins were concentrated in the 80% EtOH fraction (**Figure 1**).

### 2.6 Characterization and quantification of phenolic compounds

Flavonoids and phenolic acids were analyzed by HPLC-DAD, according to the method described in Hamdi et al, 2017. It was used a Jasco-LC-Net II ADC (Jasco, Madrid, Spain) liquid chromatograph system equipped with quaternary pump, PU 1580 and diode array detector, MD-2018 Plus. Phenolic compounds were separated by using a MEDITERRANEA SEA18 reverse-phase analytical column (25 cm length x 4.6 μm i.d., 5μm particle size; Teknokroma, Barcelona, Spain). The gradient profile for the separation of phenolics was formed using solvent A (water with 1% formic acid) and solvent B (acetonitrile with 1% formic acid) in the following program: the proportion of B was increased from 0% B to 20% B for the first 20 min, then to 21% B over the next 8 min, maintained at 21% B for 2 min, then to 30% B over the next 10 min, and to 100% over the next 5 min, maintained at 100% B for 5 min and finally returned to the initial conditions over the next 5 min. The flow rate was 1 mL/min and the column temperature was 30°C. Spectra from all peaks were recorded in the 200-600 nm range and the chromatograms were acquired at 360 nm for flavonoids and 280 for phenolic acids. Quantitation was by integration of peak-areas at selected wavelengths, with reference to calibrations made using known amounts of pure compounds. The flavonoid profiles of both standard and aqueous extracts from asparagus fronds are shown in (**Supplementary Files 1–3**)

### 2.7 Characterization and quantification of saponins

The method for saponin analysis was previosuly developed by our research group and it is described in detail in Vazquez-Castilla et al. (2013) Briefly, a HPLC Waters Alliance (Manchester, UK) system equipped with separation module 2695, diode array detector PDA 996 and Acquity QDA detector. Saponins were separated by using a MEDITERRANEAN SEA18 reverse-phase analytical column (25 cm length × 4.6 mm id., 5 *μ*m particle size; Teknokroma, Barcelona). An elution gradient was used with solvent A (water with 1% formic acid) and B (acetonitrile with 1% formic acid): 0 to 30 min, 20% B; 30 to 60 min, linear gradient to 30% B; 60 to 70 min linear gradient to 100% B, and 70–80 min, linear gradient 20% B. The saponins were detected using an online connected quadrupole mass analyzer (ZMD4, Micromass, Waters, Inc., Manchester,U.K.). Electrospray ionization (ESI) mass spectra were obtained at ionization energies of 50 and 100 V (negative mode) and 50 V (positive mode) with scans from *m/z* 200 to 1200. Capillary voltage was 3 kV, the desolvation temperature was 200°C, source temperature 100°C, and extractor voltage 12 V. The flow rate was kept at 1 mL/min and a split ratio of 5:1 for each analysis.

Quantitative analysis was made by using the external standard method as described by Vazquez-Castilla et al, 2013. For each standard, the selected ion chromatogram corresponding to its molecular ion in negative mode at 100 V was integrated and the peak area was plotted against concentration and subjected to regression analysis. The saponin profiles of both standard and aqueous extracts from asparagus roots are shown in **Supplementary Files 4, 5**).

### 2.8 Statistical analysis

Results were expressed as mean values ± standard deviations. To assess for differences in the composition and functional characteristics between the different asparagus samples, a multiple sample comparison was performed using the Statgraphics Plus program version 2.1. Multivariate analysis of variance (ANOVA), followed by Duncan’s multiple comparison test, was performed to contrast the groups. The level of significance used was P < 0.05.

## 3 Results and discussion

### 3.1 Optimization of methods for extraction of phytochemicals from asparagus cultivation by-products

The extraction of phytochemicals from asparagus by-products was carried out from fresh material, which is a clear advantage, particularly in the case of plant materials like asparagus, in which the water contents are higher than 70%. The drying step currently represents one of the largest items in production costs of plant extracts, especially with the rising price of fossil fuels. Moreover, drying processes may negatively influence the phytochemical quality of these asparagus co-products and, therefore, their additional value. In previous works, the extraction of phenolics and saponins from asparagus spears was performed by using ethanol: water solutions that solubilize total contents of these phytochemicals present in asparagus tissues (Fuentes-Alventosa et al, 2007; Vazquez-Castilla et al, 2013). Later on, it was developed a process that was scaled up to the pilot plant level and is protected under patent (Guillén-Bejarano et al, 2012). In the present work, a series of preliminary tests have been carried out prior to achieve the most effective method for obtaining bioactive compounds from asparagus fronds and roots. The design of the experiment has consisted of:

1. Compare the extractive capacity of ethanol and water, confirming that the efficiency of the latter is similar to that of ethanol.
2. Establish the optimum temperature for the treatment of roots and fronds at 121°C, after verifying that lower temperatures, 80-100°C, are not sufficient to solubilize all the compounds of interest.
3. Determine that the use of a closed reactor (laboratory or industrial autoclave) is more efficient than extraction in an open thermostatic bath.
4. Set the optimum extraction time at 2 hours, after having tested periods of between 30 minutes and 4 hours.

The optimized method for the extraction of asparagus phytochemicals consisted of the treatment of asparagus by-product with water, as extraction solvent, in a ratio of 1:2 solid: liquid (w/v), at 121°C, for 2 h. After the hydrothermal treatment, the recoveries of flavonoids and saponins in the aqueous extract were 56% and 61% respect to the contents obtained in the ethanolic extracts. It is remarkable that the fibrous residue obtained after separating the aqueous extract, by filtration, which constitutes the asparagus bioactive fibre, also contained significant quantities of flavonoids and saponins that remained linked to the fibre during the hydro-thermal treatment above described. The recoveries of flavonoids and saponins, calculated as the sum from the aqueous extract and fibrous residue, were about 80% from both types of phytochemicals (Fuentes-Alventosa et al, 2013).

### 3.2 Phytochemical composition of asparagus fronds

**Figure 2:**
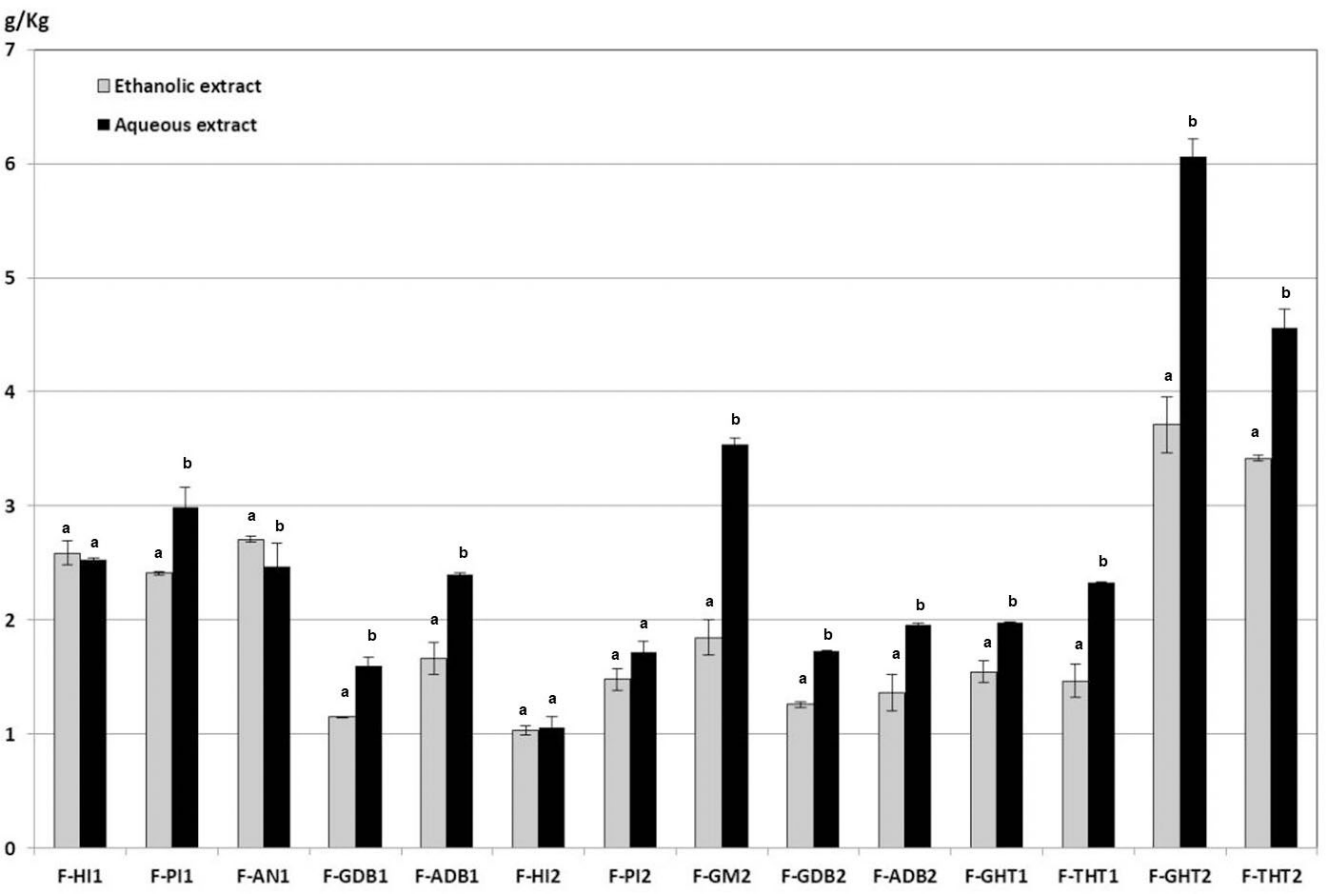
Flavonoid contents of ethanolic and aqueous extracts from asparagus fronds. Means within each sample on the x-axis bearing the same low case letter are not significantly different at 5% level, as determined by the Duncan multiple range test.

**Figure 3:**
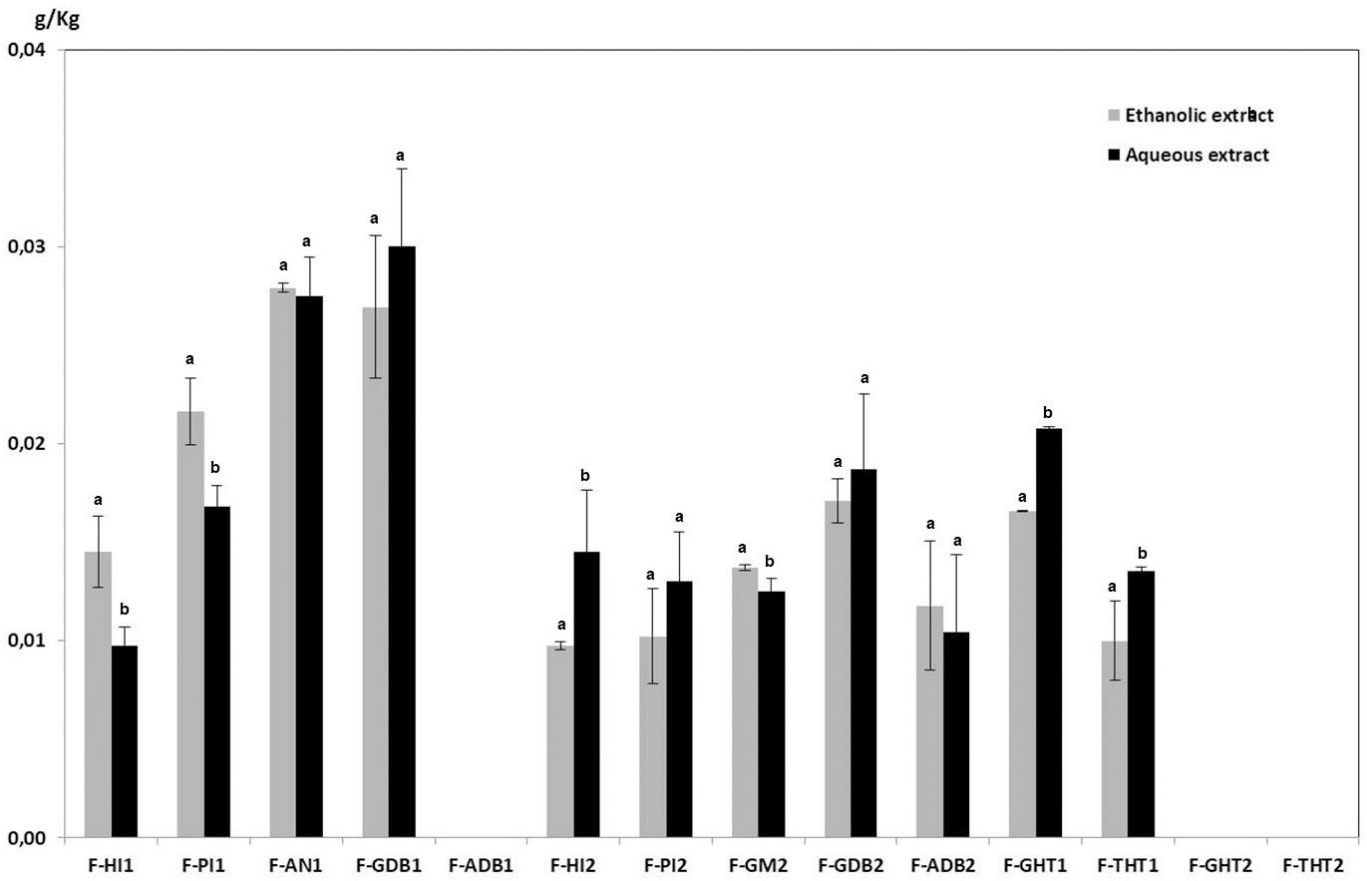
Saponin contents of ethanolic and aqueous extracts from asparagus fronds. Means within each sample on the x-axis bearing the same low case letter are not significantly different at 5% level, as determined by the Duncan multiple range test.

In the present work, a comparative analysis of phytochemical composition from asparagus fronds extracted with hot water and with 80% ethanol has been carried out. Flavonoids were main phytochemicals from aerial part of asparagus plants, but it has been detected that they are accompanied by minor quantities of saponins. It was observed that, in general, the samples from Huétor-Tájar (Granada) contained the highest amounts of flavonoids and the lowest of saponins. Similar qualitative composition of flavonoids and saponins were found in the aqueous and ethanolic extracts from fronds, as it can be observed in **Figures 2**, **3.** However, it is remarkable that total amounts of flavonoids and saponins extracted with water were significantly higher than those solubilized with ethanol solutions. Average flavonoid content was 2.637 ± 0.014 g/Kg fresh weight for hot water extraction and 1.997 ± 0.017 g/Kg in the case of ethanolic extracts. Saponin content in fronds was much lower than that of flavonoids and no significant differences were found between water and ethanol extraction (0.014 ± 0.001 g/Kg and 0.013 ± 0.002 g/Kg respectively). From this comparative analysis, it can be concluded that the extraction with hot water is the most adequate solvent for the solubilization of phytochemicals from asparagus by-products. Therefore, the presentation and discussion of the results will be focused on the aqueous extracts in the next sections of the article.

The moisture of the frond samples was about 70%, so the average content of flavonoids were 8.79 g/Kg dry weight, being the lowest value 3.52 g/kg and the highest 20.21 g/Kg, which are greater than values reported by other authors that have investigated the phytochemicals of several species of *Asparagus*. Hamdi et al. (2017) studied the phenolic profile of distinct plant parts of *Asparagus albus* and reported that leaves, where the highest quantity of phenolics was concentrated, contained 6.03 g/kg dry weight, and the amount reported by Durai Prabakaran et al. (2015) for *Asparagus racemosus* was 3.44 g/Kg.

There are scarce references about the presence of saponins in aerial parts from cultivated asparagus, as they are usually associated to the hardened basal portions of the spears, especially the white ones (Schwarzbach et al, 2006; Brueckner et al, 2010). In the present study, we have found that fronds contain flavonoids as major components, but they are accompanied by saponins as minor components. The average value of saponins from fronds was much lower than that of flavonoids (32.50 mg/Kg dried sample), being the lowest value 13.75 and the highest 45.82. It has been proposed that these phytochemiclals may also contribute to the high antioxidant capacity that owns asparagus aerial parts, which is usually associated to the phenolic compounds. Other therapeutics properties that have been described for extracts from the aerial parts of wild asparagus species and that can be partially attributed to saponins are antibacterial activity (Ntsoelinyane and Mashele, 2014), prevention of breast cancer (Mustarichie et al., 2011) and gastroduodenal diseases (Shah et al., 2014). In this context, Hamdi et al. (2017) stated that the leaves from *A. albus* contained much more saponins (32 g/Kg dried weight) than flavonoids (6 g/Kg dried weight). However, it should be noted that that they are wild species that grow under different ecological conditions and environmental stresses, which is related to the increase in the synthesis of secondary metabolites as a defense system. In fact, the use of many *Asparagus* plants, native to the Mediterranean region, in traditional medicine is based in their high levels of bioactive compounds, mainly phenolics and saponins (Al-Snafi, 2015).

Takacs-Hajos and Zsombik (2015) reported that if aerial parts of asparagus plants are trimmed when the stems and leaves are still green, their content of flavonoids is very high, between 4 and 7 times higher than that of the spears. Comparing the results of the present work with those from our previous investigations on asparagus spears, we can also conclude that asparagus fronds are much richer in flavonoids than the edible portion of the plant. In previous works, it was investigated the phytochemical composition of nearly 100 distinct genotypes of green asparagus, including the most cultivated commercial hybrids that have been developed in recent years by major international asparagus programs and different genotypes of *triguero* spears, autochthonous of Huétor-Tájar (Granada). The results revealed that the last contained greater amounts of antioxidants, mainly flavonoids (0.520 g/Kg fresh weight), than the former (0.429 g/Kg fresh weight). As it has been mentioned above, the average content of flavonoids from the fronds investigated in this work was 2.64 g/Kg fresh weight which is up to 5-6 times higher than that of the spears.

In the present work, the detailed composition of flavonoid complement has also been determined, as it can be observed in **Table 2**. In consonance with previous studies of flavonoid composition from asparagus aerial parts, it was established that rutin was the main flavonoid in all the samples investigated, representing about 94% of the total flavonoid complement. These results are in consonance with several authors that have previously reported that rutin was the most representative functional component of asparagus (Tsushida et al, 1994; Chin et al., 2002; Maeda et al., 2005). It has also been described that rutin has interesting biological activities, such as anti-inflammatory and antihypertensive (Hellerstein et al., 1951; Selloum et al., 2003).

**Table 2:** Flavonoid composition of aqueous extracts from asparagus fronds (g/Kg).

	Q-3-R-Rut	Q-3-G-Rut	IR-3-R-Rut	Rutin	IR-3-G-Rut	Nicotiflorin		Narcissin	IR-3-G	Total
HI1	0.012 ± 0.001 ^b^	0.029 ± 0.005 ^b^	0.026 ± 0.001 ^g^	2.538 ± 0.001 ^d^		0.011 ± 0.000 ^a^		0.031 ± 0.002 ^bc^		2.532 ± 0.014 ^d^
PI1			0.019 ± 0.007^de^	2.890 ± 0.165 ^e^		0.025 ± 0.001 ^bcd^		0.035 ± 0.005 ^cd^	0.020 ± 0.000 ^ab^	2.989 ± 0.172 ^e^
AN1		0.016 ± 0.002 ^a^	0.027 ± 0.007 ^g^	2.343 ± 0.199 ^d^		0.026 ± 0.001 ^cd^		0.031 ± 0.002 ^bc^	0.023 ± 0.001b^cd^	2.467 ± 0.204 ^d^
GDB1	0.001 ± 0.000 a		0.013 ± 0.002 ^bcd^	1.508 ± 0.007 ^b^		0.018 ± 0.001 ^abc^		0.026 ± 0.002 ^b^	0.026 ± 0.001^cde^	1.600 ± 0.069 ^b^
ADB1			0.015 ± 0.001 ^de^	2.307 ± 0.001 ^d^		0.022 ± 0.000 ^bcd^		0.038 ± 0.000^cd^	0.018 ± 0.001 ^a^	2.401 ± 0.012 ^d^
HI2				1.012 ± 0.100 ^a^		0.104 ± 0.000 ^a^		0.015 ± 0.001 ^a^	0.019 ± 0.001 ^a^	1.057 ± 0.098 ^a^
PI2			0.008 ± 0.000 ^a^	1.631 ± 0.095 ^bc^		0.018 ± 0.001 ^abc^		0.043 ± 0.003 ^de^	0.018 ± 0.001 ^a^	1.718 ± 0.101 ^bc^
GM2			0.028 ± 0.004 ^g^	3.346 ± 0.062 ^f^	0.055 ± 0.000 ^b^	0.038 ± 0.000 ^d^		0.059 ± 0.000 ^f^	0.019 ± 0.001 ^a^	3.538 ± 0.060 ^f^
GDB2			0.011 ± 0.000 ^bcd^	1.539 ± 0.002 ^b^	0.098 ± 0.001 ^c^	0.016 ± 0.001 ^ab^		0.034 ± 0.002 ^bc^	0.030 ± 0.001 ^e^	1.728 ± 0.003 ^bc^
ADB2			0.010 ± 0.000 ^bc^	1.713 ± 0.007 ^bc^	0.106 ± 0.014 ^c^	0.025 ± 0.001 ^bcd^		0.050 ± 0.008 ^ef^	0.051 ± 0.002 ^f^	1.955 ± 0.023 ^c^
GHT1			0.020 ± 0.001 ^ef^	1.873 ± 0.005 ^c^		0.031 ± 0.004 ^d^		0.031 ± 0.001^bc^	0.026 ± 0.001 ^de^	1.982 ± 0.002 ^c^
THT1			0.024 ± 0.000 ^f^	2.251 ± 0.004 ^d^		0.018 ± 0.001 ^abc^		0.014 ± 0.003 ^a^	0.022 ± 0.001 ^abc^	2.329 ± 0.001 ^d^
GHT2			0.028 ± 0.002 ^g^	5.740 ± 0.159 ^h^	0.060 ± 0.006 ^b^	0.080 ± 0.004 ^f^		0.155 ± 0.008 ^g^		6.062 ± 0.156 ^h^
THT2			0.021 ± 0.000 ^ef^	4.262 ± 0.157 ^g^	0.043 ± 0.004 ^a^	0.058 ± 0.003 ^e^		0.176 ± 0.006 ^h^		4.560 ± 0.161 ^g^

Means within a column bearing the same letter are not significantly different at 5% level as determined by the Duncan multiple range test

Most recently it has been proposed that non-usable parts of asparagus plants should be promoted as a rutin source, and cultivation techniques to increase the rutin content of asparagus may be developed in the future. Hence, Motoki et al. (2012) investigated the distribution of rutin and protodioscin among the different parts of asparagus plants and concluded that large amounts of rutin was concentrated in both aerial and aboveground parts. They determined rutin in each of the different parts that conforms asparagus fronds: cladophylls, branchs, stems, flowers, fruits and seeds and quantified the highest content from cladophylls (20.46 g/Kg dry weight). The mean value of rutin, calculated from all constituents of aerial parts, would be about 6.73 g/Kg dry weight, which is equivalent to the contents quantified in the present work (8.28 g/Kg dry weight). Flavonoid profile from the samples investigated in this study revealed that rutin was accompanied by up to seven other different flavonoid glycosides derived from three distinct aglycones, quercetin, kaempferol and isorhamnetin. Qualitative composition of flavonoids was very similar to that previously described for asparagus spears (Fuentes-Alventosa et al, 2008; Jaramillo-Carmona et al, 2019), but there were several differences related to the relative percentage of rutin, which was significantly higher in the aerial parts (94%) than in the edible portion (74%). It is noteworthy that despite rutin is clearly the major component, the other quercetin derivatives were not detected in the majority of cases and they were present, in very low amounts, only in 2-3 samples. They were also quantified significant amounts of nicotiflorin (kaempferol-3-O-rutinoside) and isorhamnetin (IR) derivatives: IR-3-O-rutinoside or narcissin, IR-3-O-rhamnosyl-rutinoside, IR-3-O-glucosyl-rutinoside and IR-3-O-glucoside. They may contribute to enhance and diversify the potential applications of these flavonoid extracts, since in addition to the properties common to all flavonoids, such as high antioxidant capacity, each of the individual compounds provide more specific activities that further increase the interest of these bioactive extracts. Hence, Jaramillo et al. (2010) demonstrated that isorhamnetin inhibits cell growth and induces cytotoxicity in f human colon cancer cells, which may have clinical significance with therapeutic and chemopreventive capabilities.

Saponin composition from asparagus fronds was also investigated and the results are summarized in **Table 3**. Protodioscin was the major saponin present in all the investigated samples, being the unique detected in some cases. In addition, there were quantified significant amounts of three other saponins, which had been previously identified in triguero asparagus spears (Vazquez-Castilla et al, 2013).

**Table 3:** Saponin composition of aqueous extracts from asparagus fronds (g/Kg).

	HTSAP1	HTSAP4	PROTODIOSCIN	HTSAP11	Total
HI1					n.d.
PI1			0.012 ± 0.000 ^b^	0.005 ± 0.001 ^a^	0.017 ± 0.001 ^c^
AN1			0.028 ± 0.000 ^e^		0.028 ± 0.002 ^e^
GDB1			0.030 ± 0.001 ^e^		0.030 ± 0.002 ^e^
ADB1					n.d.
HI2			0.010 ± 0.000 ^ab^	0.004 ± 0.000 ^a^	0.014 ± 0.001 ^b^
PI2	0.028 ± 0.001 ^b^		0.104 ± 0.001 ^f^		0.013 ± 0.000 ^b^
GM2			0.012 ± 0.001 ^b^		0.012 ± 0.001 ^ab^
GDB2	0.015 ± 0.000 ^a^	0.003 ± 0.001 ^a^	0.016 ± 0.001 ^c^		0.020 ± 0.002 ^cd^
ADB2			0.010 ± 0.000 ^ab^		0.010 ± 0.002 ^a^
GHT1			0.021 ± 0.000 ^d^		0.021 ± 0.000 ^d^
THT1		0.006 ± 0.000 ^b^	0.007 ± 0.000 ^a^		0.013 ± 0.000 ^b^
GHT2					n.d.
THT2					n.d.

Means within a column bearing the same letter are not significantly different at 5% level as determined by the Duncan multiple range testn.d., no detected.

Analysis of the 14 different frond samples included in this study has provided a large number of data that have been statistically treated to determine the influence of several factors on the phytochemical composition of asparagus fronds. **Table 4** summarizes flavonoid contents of five asparagus varieties, from four locations of Cadiz and one in Granada. They are: Herkolim var.-IFAPA (H-I), Primens var. –IFAPA (HI), Alamo var.-La Negra (A-N), Grande var.–Doña Blanca (G-DB), Aticus var.-Doña Blanca (A-DB), Grande var.-Manrique (G-M), Grande var.-Huétor Tájar (G-HT) and Triguero-Huétor Tájar (T-HT). Samples were collected at two different dates, with the exception of two of them A-N that was only available in the first point and G-M in the second one. In first term, we analyzed the differences between summer and autumn samples and found that in most samples from Cádiz, there was a significant decrease of flavonoids during the summer, with the exception of G-DB, where flavonoid contents did not change. The tendency was just the opposite in the samples from Granada, where it was observed a high increase of flavonoids in autumn. This fact may be linked to the special cultivation system of this area where, in addition to spring harvest, a second collect is carried out at the end of summer. This causes greater stress to the plants that defend themselves by increasing the synthesis of secondary metabolites, among which the flavonoids stand out. Regarding the composition of distinct varieties, no significant differences were found among them. If we focus on the Grande variety, which is the only one for which we have data from different growing areas, it can be established that the location does influence flavonoid composition of asparagus fronds, as confirmed by multifactorial analysis of variance.

**Table 4:** Influence of genetic and environmental factors in flavonoid contents from asparagus fronds (g/Kg).

AQUEOUS EXTRACT								
	June-July				September-October			
F-HI	2.532	±	0.002	^b D^	1.057	±	0.001	^a A^
F-PI	2.989	±	0.172	^b E^	1.718	±	0.101	^a B^
F-AN	2.467	±	0.204	^D^				
F-GM					3.538	±	0.006	^C^
F-GDB	1.600	±	0.081	^a A^	1.728	±	0.003	^a B^
F-ADB	2.401	±	0.012	^b C^	1.955	±	0.306	^a B^
F-GHT	1.982	±	0.000	^a B^	6.062	±	0.142	^b E^
F-THT	2.329	±	0.001	^a C^	4.560	±	0.172	^b D^

Means within a row bearing the same low case letter are not significantly different at 5% level, as determined by the Duncan multiple range test. Means within a column bearing the same capital letter are not significantly different at 5% level, as determined by the Duncan multiple range test.

Results evidenced that asparagus fronds are a very good source of flavonoids, mainly rutin, whit a high antioxidant activity. These phytochemicals are soluble in water and can be easily obtained in form of aqueous extracts with an average yield of 1.05% based on the fresh wright of fronds and a flavonoid richness of 17%. The antioxidant extracts partially purified and enriched in flavonoids can be used as bioactive ingredient in several prepared foods. In the food ingredients market there is a growing demand for these extracts of plant polyphenols, some of which, such as those from rosemary and tea, have already been approved by the European Union as a safe and natural antioxidant for the preservation of different types of food, including meat products, fish, cookies and dairy. The value of these natural antioxidants lies not only in their ability to maintain the organoleptic and nutritional quality of the matrices to which they are added, by preventing oxidative deterioration processes, but also in their antimicrobial activity that contributes to preserving microbiological quality. We have also carried out preliminary assays on the use of our extracts from asparagus fronds in the preparation of cured meat products and the results are promising.

### 3.3 Phytochemical composition from asparagus roots

The crown and roots from old asparagus plants that are no longer productive can make up to 85% of the plant’s total weight and their elimination from the fields represent a considerable problem, as it has been explained above. However, the allelopathic substances that contain are at the same time bioactive compounds that are worth recovering for the preparation of bioactive extracts with great functional potential. Hence, asparagus roots are one of the best sources of plant steroidal saponins that have hepatoprotective, antiinflammatory, antibacterial, and antiaging activities, among others. Some patented processes for obtaining saponin extracts can be found in bibliography (Ordaz-Trinidad et al, 2016) and most of them implied the use of mixtures of water:organic solvents (Jing, 2012; Su, 2012).

As explained above for the extraction of phytochemicals from the aerial parts, the extraction of saponins and phenolic compounds from roots was as effective, or even more effective, with hot water as with ethanolic solutions. Previous studies on obtaining bioactive extracts from *A. albus* roots in the forms that are traditionally consumed in Tunisia, such as infusion and decoction, also revealed that saponin yields were more than two times higher in the aqueous extracts than in the ethanolic ones (Hamdi et al, 2017).

**Figure 4:**
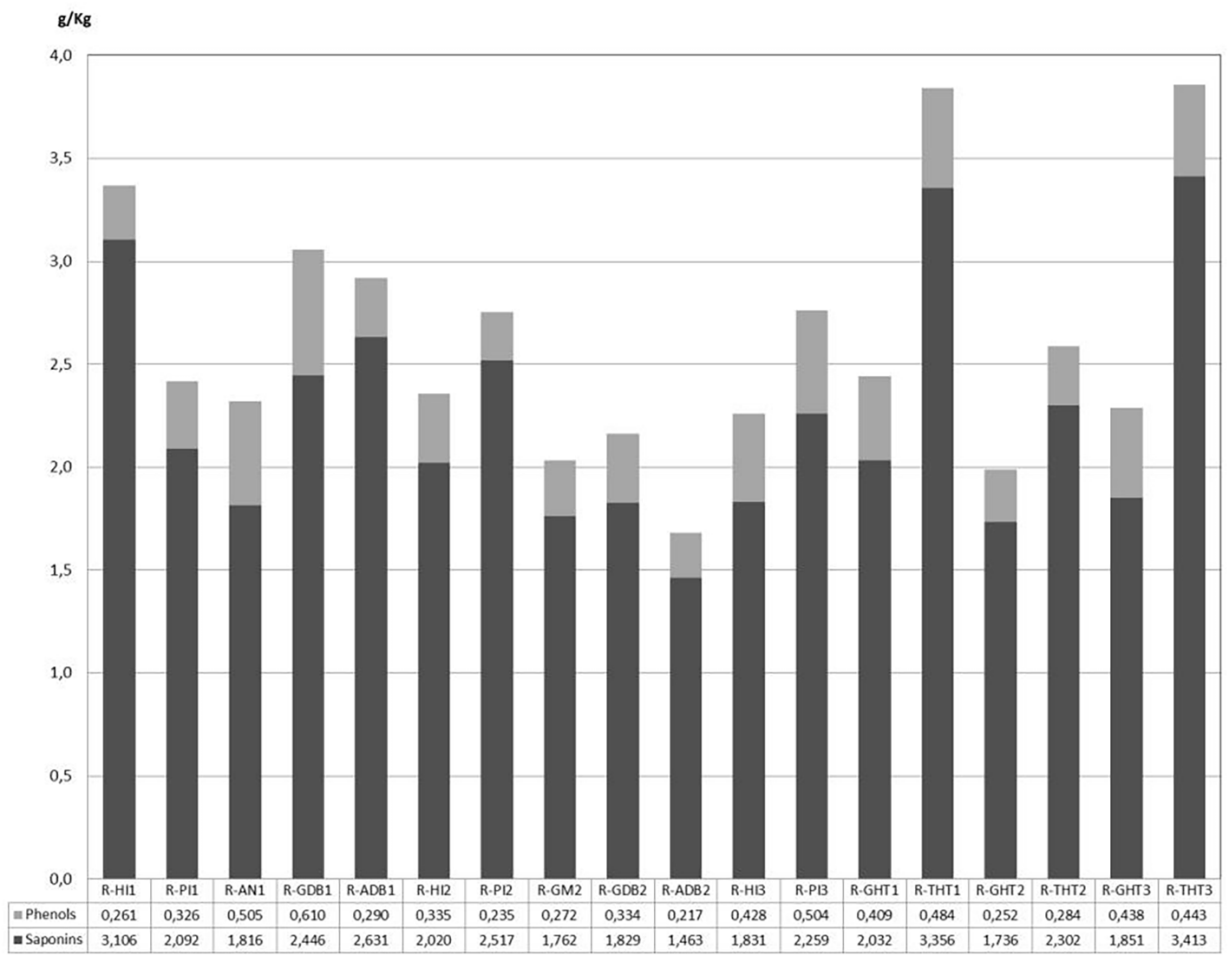
Saponins and phenolic acids contents of aqueous extracts from asparagus root.

In the present work, aqueous treatment of roots allowed obtaining an extract with a richness of 50% saponins. Average content of saponins from root samples was 2.25 g/Kg of fresh weight and, since the root moisture is 60%, the content referred to dry weight was 5.62 g/kg. In addition to saponins, significant quantities of hydroxycinnamic acids were quantified from asparagus roots. **Figure 4** summarize the contents of phenolic acids and saponins from the 18 samples of roots investigated in this study.

Total saponin contents varied between 1.46 and 3.41 g/Kg fresh weight, and they were accompanied by minor quantities of phenols (217 - 610 mg/Kg fresh weight). Composition analysis by HPLC allowed the detection of 13 different saponins, 7 of which (HTSAP1, HTSAP2, HTSAP3, Protodioscin, HTSAP11, HTSAP4, HTSAP5) had been identified in our previous works on phytochemical composition of triguero asparagus from Huétor-Tájar (Vazquez-Castilla et al, 2013). The 6 novel saponins found in asparagus roots (ARSAP) were identified by their retention time, molecular weight, and fragmentation pathway and their tentative structures are shown in **Table 5**.

**Table 5:** Tentative structures of the new saponins from asparagus roots, identified by HPLC-MS (±).

Molecular ion (m/z)					Ion fragmentation	
Saponin	Rt^a^	MW^b^	Negative ion	Positive ion	Negative mode	Positive mode
**ARSAP1**	22.44	1094	1093[M-H]^-^	1117[M+Na]^+^	(42)1051-Hex^c^ 889-Pen757-Hex595-Hex433	[+Na-H_2_O]1077-Hex915-Pen783-Hex621-Hex+(42)417
**ARSAP2**	27.71	890	889 [M-H]^-^	913[M+Na]^+^	Pen^d^757-Hex595-Hex433	[+Na-H_2_O]873-Pen741-Hex579- Hex417
**ARSAP3**	28.27	1064	1063[M-H]^-^	1087[M+Na]^+^	(42)1021-Pen889-Pen757-Hex595-Hex433	[+Na-H_2_O]1047-Hex885-Pen753-Pen621-Hex+(42)417
**ARSAP4**	44.91	872	871[M-H]^-^	895[M+Na]^+^	Pen739-Hex577	[+Na+H]873-Hex711-Pen579-Hex417
**ARSAP5**	46.57	740	739M-H]^-^	763[M+Na]^+^	Hex577	[+Na+H]741-Hex579-Hex417
**ARSAP6**	47.62	842	841[M-H]^-^	865[M+Na]^+^	Pen709-Pen577	[+Na+H]843-Pen711-Pen579-Hex417

^a^Rt, retention time; ^b^MW, molecular weight; ^c^Pen, pentose; ^d^Hex, hexose.

The compositions of each saponin present in asparagus roots and the total contents are listed in **Table 6**. It is remarkable that the saponins previously quantified in significant amounts in the spears are also the most abundant in the roots of samples from Cadiz. However, the new saponins identified in this study represent up to 50% total saponin content in the roots from Granada. In fact, 4 of them (ARSAP1, ARSAP2, ARSAP3 AND ARSAP6) are only present in samples of triguero asparagus from Huétor Tájar (THT). It has been reported that protodiscin is the major saponin in cultivated asparagus and its concentrations vary significantly among the different organs of the plant (Wang et al, 2003; Lee et al, 2010; Maeda et al, 2012). Hence, asparagus edible portion contains much lower amounts of saponins than other plant organs, such as fruits, rhizomes and roots but the final portion of the spear that is discarded during processing, accumulates up to 600 mg saponins/Kg of raw material, worth recovering to obtain bioactive extracts, as we have previously reported (Fuentes-Alventosa et al, 2013). Our results are in consonance with those from Motoki et al. (2019), who described that protodioscin was concentrated in the underground parts, finding the highest contents in buds (7.51 g/Kg dried weight) and rhizomes (3.02 g/Kg) and the lowest in fronds (0.032 g/Kg). Average contents of the samples included in the current study were very similar, quantifying up to 100 times more saponins in roots (**5.62 g/Kg dried weight**) than in the aerial part (**0.044 g/Kg**).

**Table 6:** Saponin composition of aqueous extracts from asparagus roots (g/Kg fresh weight).

	HTSAP1	ARSAP1	HTSAP2	HTSAP3	PROTOD	ARSAP2	HTSAP11	ARSAP3	HTSAP4		HTSAP5	ARSAP4	ARSAP5	ARSAP6	TOTAL
HI1	0.837 ± 0.127 ^f^		0.926 ± 0.043 ^g^	t	0.184 ± 0.011 ^de^		0.252 ± 0.002 ^f^		0.299 ± 0.027 ^g^	0.233 ± 0.03 ^cd^		0.248 ± 0.007 ^ef^	0.128 ± 0.011 ^cd^		3.106 ± 0.172 ^g^
PI1	0.517 ± 0.035 ^b^		0.664 ± 0.081 ^def^		0.052 ± 0.015 ^abc^		0.083 ± 0.006 ^cd^		0.206 ± 0.02 ^e^	0.177 ± 0.01 ^bc^		0.162 ± 0.02 ^cde^	0.232 ± 0.00 ^fg^		2.092 ± 0.073 ^bcde^
AN1	0.585 ± 0.156 ^bcd^		0.719 ± 0.073 ^ef^		0.153 ± 0.02 ^d^				0.114 ± 0.02 ^b^	0.246 ± 0.04 ^cd^					1.816 ± 0.351 ^ab^
GDB1	0.598 ± 0.132 ^bcd^		0.661 ± 0.145 ^def^		0.233 ± 0.023 ^fg^		0.096 ± 0.025 ^de^		0.118 ± 0.003 ^bc^	0.437 ± 0.064 ^g^		0.151 ± 0.036 ^bcd^	0.092 ± 0.044 ^bc^		2.446 ± 0.313 ^def^
ADB1	0.788 ± 0.083 ^ef^		0.805 ± 0.022 ^fg^		0.251 ± 0.043 ^g^		0.257 ± 0.000 ^f^		0.203 ± 0.014 ^e^	0.135 ± 0.001 ^ab^		0.128 ± 0.010 ^abc^	0.064 ± 0.020 ^ab^		2.631 ± 0.019 ^f^
HI2	0.768 ± 0.001 ^ef^		0.685 ± 0.073 ^ef^		0.036 ± 0.022 ^ab^		0.067 ± 0.004 ^bc^		0.251 ± 0.031 ^f^	0.131 ± 0.031 ^ab^		0.043 ± 0.008 ^a^	0.032 ± 0.013 a		2.014 ± 0.156 ^bcd^
PI2	0.537 ± 0.071 ^bcd^		0.867 ± 0.010 ^g^		0.199 ± 0.026 ^ef^		0.104 ± 0.006 ^de^		0.273 ± 0.037 ^fg^	0.269 ± 0.056 ^de^		0.134 ± 0.017 ^bc^	0.134 ± 0.063 ^cd^		2.517 ± 0.057 ^ef^
GM2	0.501 ± 0.018 ^b^		0.462 ± 0.014 ^bc^	t	0.143 ± 0.074 ^d^		0.038 ± 0.007 ^a^		0.115 ± 0.018 ^bc^	0.232 ± 0.032 ^cd^		0.162 ± 0.062 ^cde^	0.107 ± 0.018 ^bc^		1.761 ± 0.013 ^ab^
GDB2	0.800 ± 0.004 ^f^		0.615 ± 0.033 ^de^	0.026 ± 0.004 ^a^	0.055 ± 0.016 ^bc^				0.154 ± 0.008 ^cd^	0.117 ± 0.008 ^ab^		0.062 ± 0.063 ^ab^	t		1.829 ± 0.021 ^abc^
ADB2	0.587 ± 0.091 ^bcd^		0.519 ± 0.065 ^bcd^						0.122 ± 0.012 ^bc^	0.220 ± 0.015 ^cd^		t			1.448 ± 0.182 ^a^
HI3	0.704 ± 0.028 ^def^		0.629 ± 0.020 ^de^	t	0.032 ± 0.004 ^ab^		0.108 ± 0.014 ^e^		0.172 ± 0.016 ^de^	0.084 ± 0.000 ^a^		0.075 ± 0.049 ^abc^	0.29 ± 0.018 ^a^		1.831 ± 0.107 ^abc^
PI3	0.691 ± 0.101 ^cdef^		0.710 ± 0.032 ^ef^		0.010 ± 0.005 ^a^		0.100 ± 0.010 ^de^		0.198 ± 0.023 ^e^	0.270 ± 0.024 ^de^		0.122 ± 0.008 ^abc^	0.157 ± 0.047 ^de^		2.259 ± 0.264 ^cdef^
GHT1	0.520 ± 0.079 ^b^		0.605 ± 0.050 ^cde^	t	0.070 ± 0.004 ^bc^				0.111 ± 0.010 ^b^	0.253 ± 0.007 ^cd^		0.238 ± 0.047 ^def^	0.235 ± 0.002 ^g^		2.031 ± 0.101 ^bcd^
THT1	0.534 ± 0.065 ^bc^	0.470 ± 0.011 ^c^	0.661 ± 0.143 ^def^	0.428 ± 0.098 ^c^	0.086 ± 0.016 ^c^	0.343 ± 0.017 ^b^	0.054 ± 0.001 ^b^	0.350 ± 0,037 ^b^		0.180 ± 0.009 ^bc^		0.085 ± 0.020 ^abc^	0.104 ± 0.023 ^bc^	0.063 ± 0.005 ^a^	3.356 ± 0.402 ^g^
GHT2	0.460 ± 0.041 ^ab^		0.464 ± 0.079 ^bc^	t	0.153 ± 0.021 ^d^				0.097 ± 0.008 ^b^	0.225 ± 0.002 ^cd^		0.239 ± 0.007 ^def^	0.097 ± 0.007 ^bc^		1.735 ± 0.141 ^ab^
THT2	0.303 ± 0.033 a	0.075 ± 0.013 ^a^	0.238 ± 0.033 a	0.204 ± 0.007 ^b^	0.064 ± 0.001 ^bc^	0.071 ± 0.016 ^a^	0.009 ± 0.004 ^a^	0.081 ± 0,013 ^a^	0.026 ± 0.000 ^a^	0.323 ± 0.025 ^ef^		0.438 ± 0.033 ^g^	0.187 ± 0.002 ^ef^	0.284 ± 0.009 ^b^	2.302 ± 0.182 ^def^
GHT3	0.623 ± 0.066 ^bcde^		0.378 ± 0.004 ^ab^	0.020 ± 0.004 ^a^	0.026 ± 0.016 ^ab^				0.101 ± 0.032 ^b^	0.372 ± 0.088 ^f^		0.260 ± 0.069 ^f^	0.071 ± 0.011 ^ab^		1.851 ± 0.289 ^abc^
THT3	0.521 ± 0.091 ^b^	0.284 ± 0.073 ^b^	0.379 ± 0.092 ^ab^	0.399 ± 0.067 ^c^	t	0.239 ± 0.061 ^b^		0.328 ± 0.075 ^b^		0. 092 ± 0.010 ^a^		0.566 ± 0.114 ^h^	0.191 ± 0.034 ^efg^	0.414 ± 0.007 ^c^	3.413 ± 0.385 ^g^

Means within a column bearing the same letter are not significantly different at 5% level as determined by the Duncan multiple range test

An additional difference found in our samples consisted of that while aerial parts contained almost only protodioscin, saponin profile from roots included other 12 saponins, as explained above. This differentiation of samples based on their saponin profile is very interesting both from the point of view of classifying asparagus varieties, and for the valorization of co-products, such as roots, by obtaining bioactive extracts with different functionalities.

The influence of different factors on the saponin composition from asparagus roots was analyzed by statistical analysis and their results are summarized in **Table 7**.

**Table 7:** Influence of genetic and environmental factors in saponin contents from asparagus roots (g/Kg).

AQUEOUS EXTRACT												
	June-July				September-October				December-January			
R-HI	3.106	±	0.172	^b DE^	2.014	±	0.156	^a BC^	1.831	±	0.107	^a A^
R-PI	2.092	±	0.073	^a ABC^	2.517	±	0.057	^a D^	2.259	±	0.264	^a A^
R-AN	1.816	±	0.351	^A^								
R-GM					1.761	±	0.013	^AB^				
R-GDB	2.466	±	0.313	^a BC^	1.829	±	0.021	^a B^				
R-ADB	2.631	±	0.019	^b CD^	1.448	±	0.182	^a A^				
R-GHT	2.032	±	0.101	^a AB^	1.735	±	0.141	^a AB^	1.851	±	0.289	^a A^
R-THT	3.356	±	0. 402	^ab E^	2.302	±	0.182	^a CD^	3.413	±	0.385	^b B^

Means within a row bearing the same low case letter are not significantly different at 5% level, as determined by the Duncan multiple range test.

Means within a column bearing the same capital letter are not significantly different at 5% level, as determined by the Duncan multiple range test.

It was revealed that the sampling date is the only factor that influences the saponin content, while no significant differences were found depending on the origin and variety of the samples. In general, the amount of saponins decreased from the first sampling point (June-July) to the last (December). In consonance with our results, several authors have reported that the saponin contents are significantly lower in early spring and mid-autumn than in summer, finding that there was a trend of positive correlation between saponin concentrations and maximum temperatures (Ilieva, 1994; Pecetti et al, 2006; García-Parra et al, 2018). Saponins are secondary metabolites that the plant uses as a defense mechanism, enhancing its synthesis in different stress situations related to both biotic and abiotic factors. Hence, environmental factors, such as drought and high temperatures, may cause a significant increase in saponin content. From this it can be deduced that it would be convenient to uproot those plantations that are going to be renewed just after the asparagus season, which in Spain takes place from March to June. This practice would contribute to improving the sustainability of the crop since, on the one hand, toxic allelopathic substances are removed from the soil and, on the other hand, these crop residues are transformed into co-products with high added value. Valorization of these residues is based on obtaining bioactive extracts, rich in bioactive compounds, such as saponins, with potential application in different sectors (food, nutraceuticals and agriculture).

In addition to saponins, asparagus roots contained lower quantities of phenolics, being their average valuKg fresh sampl/Kg fresh sample (min 217 and max 610). Their composition analysis by HPLC-DAD-MS revealed that caffeic acid represented more than 90% total phenolics complement and it was accompanied by minor quantities of other hydroxycinnamic acids, such as p-coummaric acid and t-ferulic acid. Results are shown in **Table 8**.

**Table 8:** Phenolic composition of aqueous extracts from asparagus roots (g/Kg).

	Caffeic acid	p-Coumaric acid	t-Ferulic acid	Total (g/Kg)
HI1	0.249 ± 0.017 ^ab^		0.013 ± 0.000 ^b^	0.262 ± 0.017 ^bc^
PI1	0.326 ± 0.018 ^c^			0.326 ± 0.018 ^d^
AN1	0.436 ± 0.005 ^e^	0.036 ± 0.002 ^ef^	0.034 ± 0.004 ^fg^	0.506 ± 0.001 ^h^
GDB1	0.538 ± 0.007 ^g^	0.033 ± 0.002 ^e^	0.040 ± 0.003 ^g^	0.610 ± 0.001 ^i^
ADB1	0.257 ± 0.012 ^b^	0.014 ± 0.001 ^ab^	0.018 ± 0.003 ^cd^	0.289 ± 0.001 ^c^
HI2	0.335 ± 0.019 ^c^			0.335 ± 0.019 ^d^
PI2	0.221 ± 0.009 ^a^		0.014 ± 0.001 ^bc^	0.235 ± 0.011 ^b^
GM2	0.220 ± 0.006 ^a^	0.025 ± 0.001 ^d^	0.026 ± 0.001 ^e^	0.271 ± 0.001 ^bc^
GDB2	0.298 ± 0.025 ^bc^	0.013 ± 0.002 ^b^	0.023 ± 0.002 ^d^	0.334 ± 0.028 ^d^
ADB2	0.216 ± 0.002 ^a^			0.217 ± 0.002 ^a^
HI3	0.429 ± 0.022 ^ef^			0.429 ± 0.022 ^ef^
PI3	0.466 ± 0.021 ^f^	0.021 ± 0.003 ^c^	0.017 ± 0.001 ^c^	0.504 ± 0.017 ^gh^
GHT1	0.409 ± 0.020 ^e^			0.409 ± 0.002 ^e^
THT1	0.413 ± 0.005 ^e^	0.059 ± 0.002 ^g^	0.013 ± 0.002 ^b^	0.485 ± 0.008 ^g^
GHT2	0.212 ± 0.010 ^a^	0.020 ± 0.002 ^c^	0.020 ± 0.002 ^d^	0.252 ± 0.016 ^b^
THT2	0.232 ± 0.005 ^a^	0.025 ± 0.000 ^d^	0.027 ± 0.001 ^e^	0.284 ± 0.001 ^c^
GHT3	0.426 ± 0.015 ^e^	0.012 ± 0.003 ^a^		0.438 ± 0.014 ^ef^
THT3	0.364 ± 0.003 ^d^	0.047 ± 0.004 ^f^	0.031 ± 0.003 ^f^	0.442 ± 0.001 ^f^

Means within a column bearing the same letter are not significantly different at 5% level as determined by the Duncan multiple range test.

Several authors have reported the presence of flavonoids, mainly quercetin (Liu and Matsubara, 2018; Zhang et al, 2019) and rutin (Motoki et al, 2019), in asparagus roots and they proposed that these flavonoids could be responsible in part for the replanting problems associated with allelopathic substances from asparagus. However, we have not detected the presence of flavonoid compounds in any of the root samples investigated in this study, which is in consonance to the results from Symes et al. (2018). These authors have already highlighted the existing controversy about the main phenolic components in asparagus root, establishing that, contrary to what had been previously published, they had found caffeic acid as a major component and had not detected the presence of rutin or any other flavonoid. Adouni et al. (2022) also reported that phenolic composition from asparagus root consisted of several hydroxycinnamic acids and derivatives, being caffeic acid the most abundant compound, whereas no flavonoids were found. These discrepancies can be explained based on the well-established fact that considerable differences can be found in the qualitative and quantitative composition of the phytochemicals present in plant organs depending on various factors, including variety, genotype, climate, growing conditions, etc.

There are several publications that point to caffeic acid and other related hydroxycinnamic acids from asparagus root as the main allelopathic substances responsible for both decay and replanting problems (Kato-Noguchi et al, 2017; Kato-Noguchi et al, 2018; Liu and Matsubara, 2018). The most recent publications on the chemical and functional characterization of the asparagus root suggest that its high caffeic acid content, which varies between 0.57 (Adouni et al, 2022), 1.03 (Symes et al, 2018) and 2.16 g/Kg of dry root (Zhang et al, 2019), make these by-products a magnificent source to obtain natural bioactive extracts with different potentialities in the field of food-health, derived from their antioxidant activity. Our results are in the intermediate range found in bibliography, with caffeic acid values ​​ranging from 0.54 and 1.34 g/Kg dry sample.

Based on these results, the proposal for the recovery and valorization of asparagus roots includes obtaining three types of extracts, partially purified and enriched in: a) saponins, b) phenols (caffeic acid) and c) fructans. Regarding the latter, we have recently published an article about the obtention of prebiotic fructans from asparagus roots (Hamdi et al, 2023), which has been positioned among the most highly accessed articles in the journal. This confirms that there is great scientific interest in these bioactive compounds with high demand in the market of natural substances, which can gradually replace those chemically synthesized ingredients and preservatives whose use is increasingly restricted by Food Safety Agencies. The asparagus fructans have been found to have similar characteristics to other commercial fructans, so it could be used as bioactive ingredients, with prebiotic activity, in different culinary preparations, providing health benefits derived from their ability to act on the intestinal flora. With the two other extracts proposed in this article (phenolics, mainly caffeic acid, and saponins), it would be completed the valorization of asparagus roots, based on recovering all the compounds of interest that contain.

The extensive research work on the recovery of asparagus by-products that we have been carrying out for years and whose results are summarized in this article, have allowed us to achieve almost complete valorization of asparagus co-products, by obtaining 4 deliverables consisting of natural extracts with great techno-functional potential in the agri-food industry. The characteristics of those extracts, some of which are already being tested as additives in the preparation of soups, breads and meat products are the following: i) antioxidant extract enriched in flavonoids, with an average yield of 10.7 g/Kg fresh fronds and a flavonoid richness of 17%; ii) saponins extract with an average yield of 10.3 g/Kg fresh root and a richness of 51%; iii) prebiotic extract with a high content of fructans, with an average yield of 175,9 g/Kg fresh root and a fructans richness of 57.9%, iv) insoluble fibre fraction with a high antioxidant capacity, derived from its phenolic content (8.3 mg/Kg) and whose average yield was 114.6 g/Kg.

## 4 Conclusions and future prospects

As a final remark on this work, we would highlight that our future research involves validating the specific uses of the different deliverables that we can design from asparagus by-products, which will have a significant scientific and social impact. The fact that these new ingredients come from plant residues that were initially destined for disposal as waste, with the enormous costs that this entails, greatly contributes to improving the sustainability of the crop, within the scope of the circular economy, providing farmers with new alternatives for the recovery of their by-products; and to the food industries a portfolio of natural ingredients of great applicability in their formulations.

Specific applications of these bioactive ingredients in different food matrices will consist of: i) addition of antioxidant extracts from asparagus fronds to bakery and meat products, to verify their effectiveness in extending their commercial life; ii) inclusion of saponins in creams, soups and juices to improve their stability and foaming capacity; iii) design of natural phytosanitary products, based on flavonoids and saponins, with potential anti-pesticide and biostimulant activity on different crops.

The use of natural extracts as bioactive ingredients has great potential, but further studies are required on the chemical compounds responsible for the technofunctional activities attributed to plant extracts. Future research will focus on the study of individual bioactive compounds, to identify which are the key molecules in the different biological activities attributed to asparagus extracts.

## Data availability statement

The raw data supporting the conclusions of this article will be made available by the authors, without undue reservation.

## Author contributions

IV has been implied in preparation of the extracts and identification of phenolics; AH has participated in preparation of samples and has been developed most work related to identification of different compounds by HPLC; RG-B has contributed to the work related to saponis; AJ-A has been more directly related to the collection of the samples and the statistical analysis. RR-A has been in charge of designing the work experiences, has been especially involved in the determination of phenols and has written the manuscript. All authors contributed to the article and approved the submitted version.

## Supplementary material

The Supplementary Material for this article can be found online at: The Supplementary Material for this article can be found online at: https://www.frontiersin.org/articles/10.3389/fpls.2023.1199436/full#supplementary-material

Supplementary 1 Chromatographic profile acquired by HPLC-DAD (360 nm) of flavonoid standards.

Supplementary 2 Chromatographic profile acquired by HPLC-DAD (360 nm) of flavonoids isolated from asparagus fronds

Supplementary 3 Typical chromatographic profile acquired by HPLC-DAD (360 nm) of flavonoid from asparagus fronds.

Supplementary 4 Chromatographic profile acquired by HPLC-MS of saponin standards.

Supplementary 5 Typical chromatographic profile acquired by HPLC-DAD-MS of saponins from asparagus roots.
