# Supplementary figures and images for: Sustainable valorization of co-products from asparagus cultivation by obtaining bioactive compounds
Source: Front Plant Sci. 2023 Jul 13;14:1199436. doi: 10.3389/fpls.2023.1199436 (PMC10373885; doi:10.3389/fpls.2023.1199436)

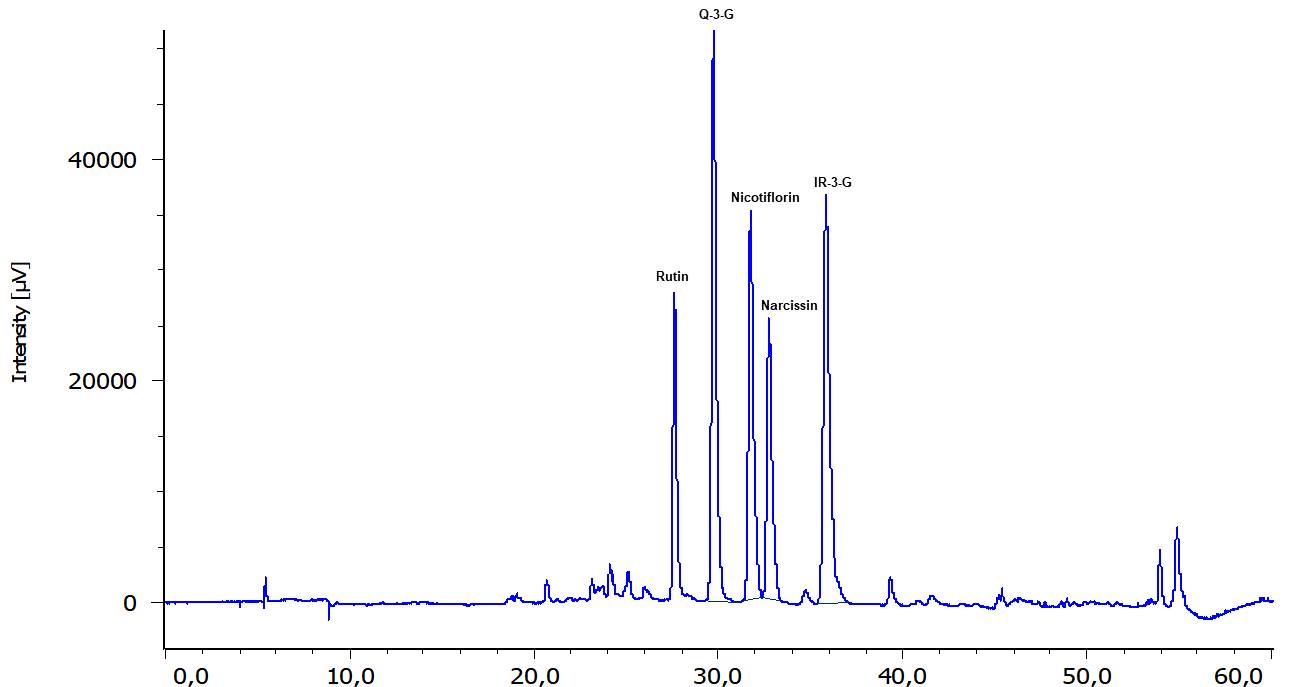

Supplement: Supplementary 1 [file Image_1.jpeg]

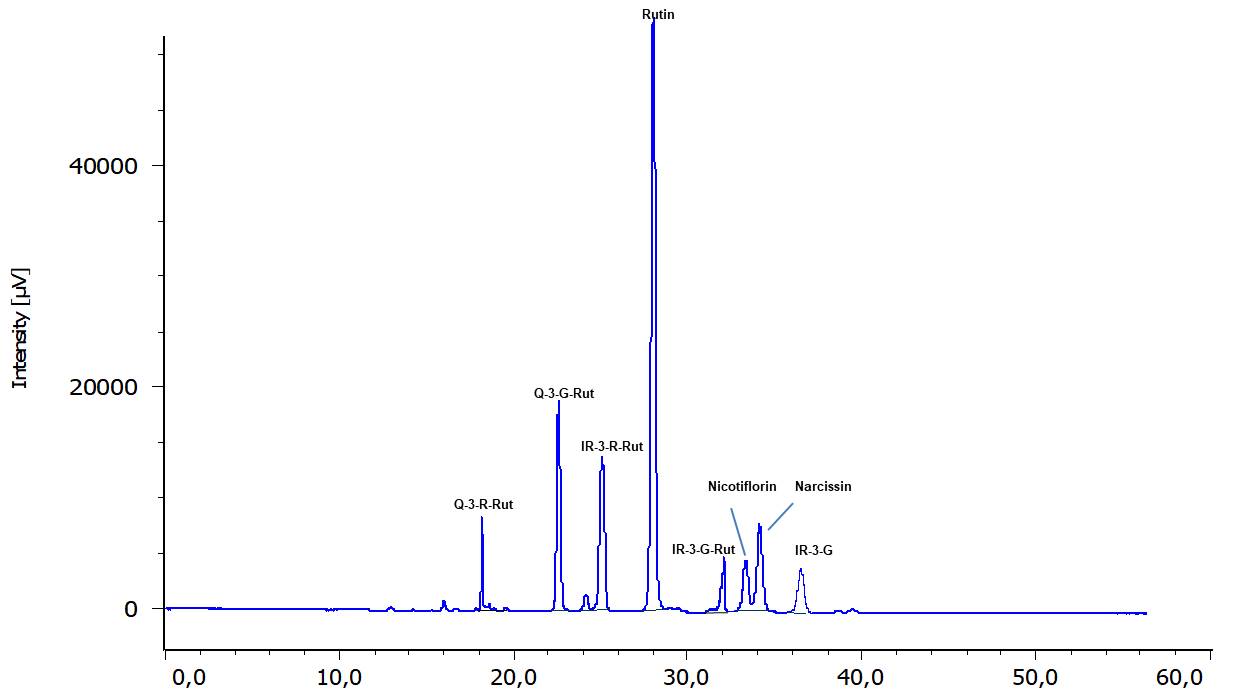

Supplement: Supplementary 2 [file Image_2.jpeg]

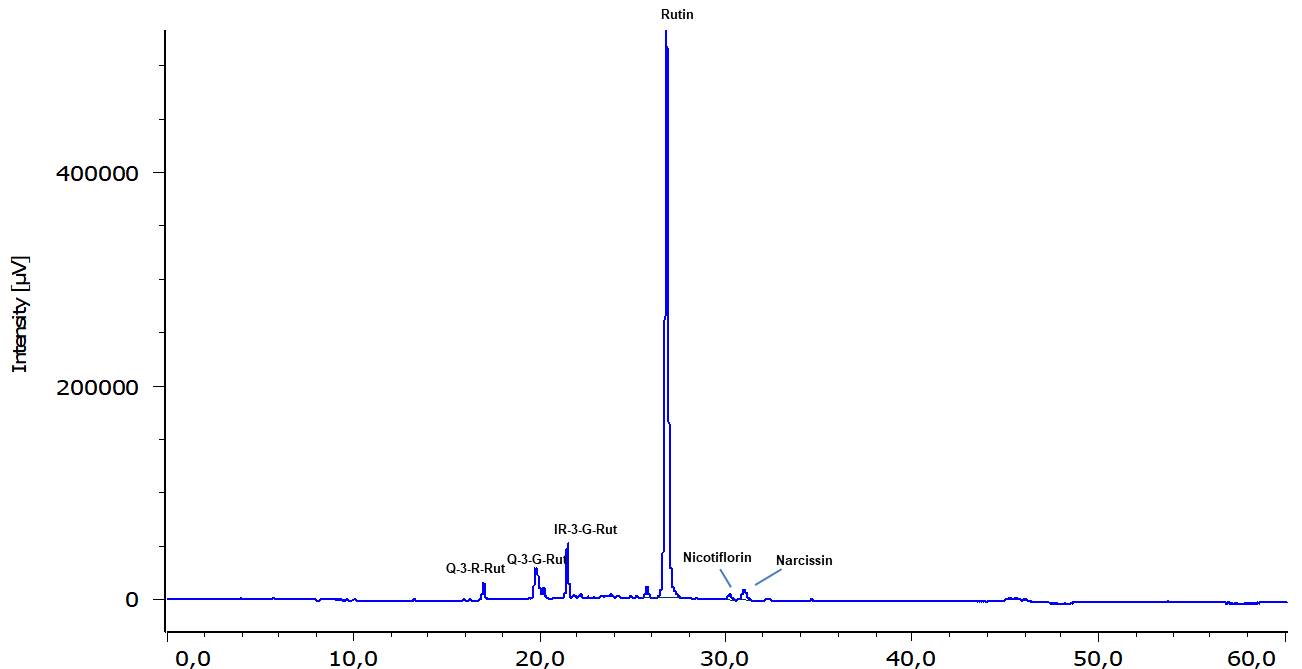

Supplement: Supplementary 3 [file Image_3.jpeg]

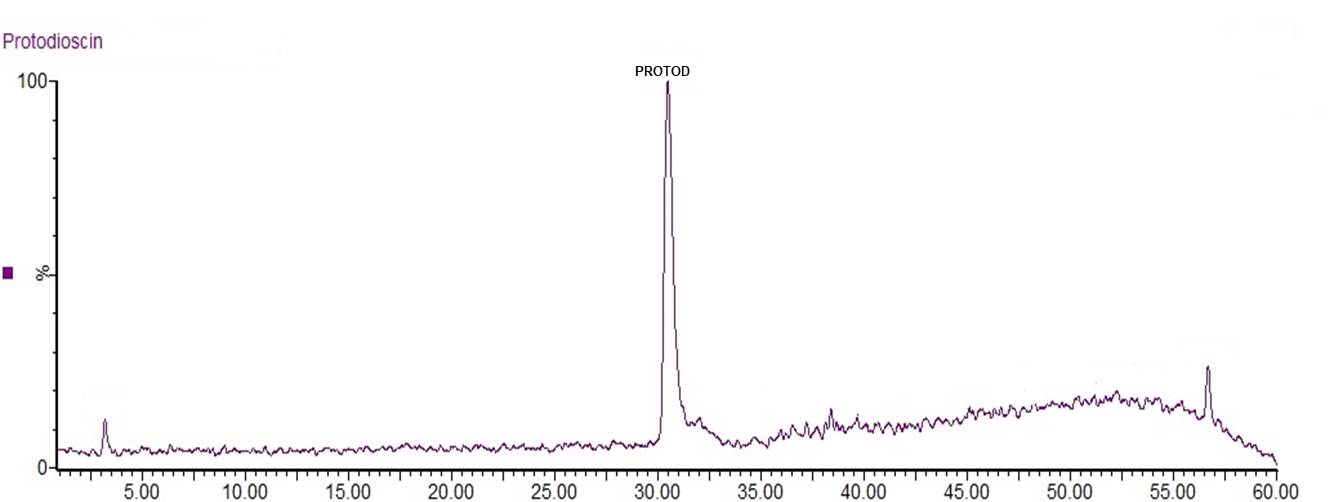

Supplement: Supplementary 4 [file Image_4.jpeg]

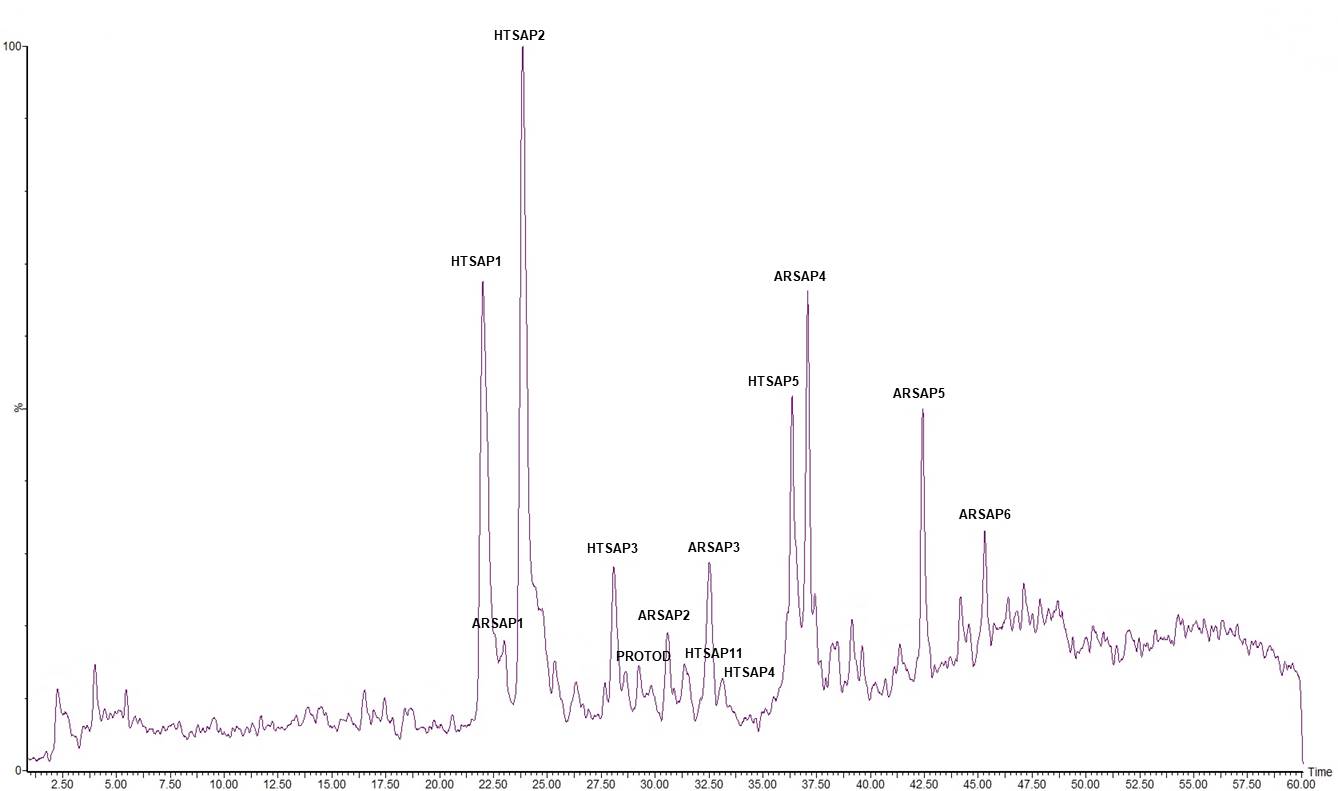

Supplement: Supplementary 5 [file Image_5.jpeg]
